# Ziyuglycoside I Inhibits the Proliferation of MDA-MB-231 Breast Carcinoma Cells through Inducing p53-Mediated G2/M Cell Cycle Arrest and Intrinsic/Extrinsic Apoptosis

**DOI:** 10.3390/ijms17111903

**Published:** 2016-11-22

**Authors:** Xue Zhu, Ke Wang, Kai Zhang, Ting Zhang, Yongxiang Yin, Fei Xu

**Affiliations:** 1 Key Laboratory of Nuclear Medicine, Ministry of Health, Jiangsu Key Laboratory of Molecular Nuclear Medicine, Jiangsu Institute of Nuclear Medicine, Wuxi 214063, China; zhuxue@jsinm.org (X.Z.); wangke@jsinm.org (K.W.); zhangkai@jsinm.org (K.Z.); 2 The Affiliated Maternity and Children Health Hospital of Nanjing Medical University, Wuxi 214002, China; zhangting040715@163.com; 3 Department of Molecular Cell Biology and Toxicology, Jiangsu Key Lab of Cancer Biomarkers, Prevention and Treatment Cancer Center, School of Public Health, Nanjing Medical University, Nanjing 210029, China; 4 Department of Pathology, the Affiliated Maternity and Children Health Hospital of Nanjing Medical University, Wuxi 214002, China; yinyongxiang0118@outlook.com; 5 Department of Laboratory Medicine, the Affiliated Maternity and Children Health Hospital of Nanjing Medical University, Wuxi 214002, China

**Keywords:** ziyuglycoside I, MDA-MB-231 cell line, G2/M phase arrest, intrinsic and extrinsic apoptosis

## Abstract

Background: Due to the aggressive clinical behavior, poor outcome, and lack of effective specific targeted therapies, triple-negative breast cancer (TNBC) has currently been recognized as one of the most malignant types of tumors. In the present study, we investigated the cytotoxic effect of ziyuglycoside I, one of the major components extracted from Chinese anti-tumor herbal *Radix Sanguisorbae*, on the TNBC cell line MDA-MB-231. Methods: The underlying molecular mechanism of the cytotoxic effect ziyuglycoside I on MDA-MB-231 cells was investigated with cell viability assay, flow cytometric analysis and Western blot. Results: Compared to normal mammary gland Hs 578Bst cells, treatment of ziyuglycoside I resulted in a significant growth inhibitory effect on MDA-MB-231 cells. Ziyuglycoside I induced the G2/M phase arrest and apoptosis of MDA-MB-231 cells in a dose-dependent manner. These effects were found to be partially mediated through the up-regulation of p53 and p21^WAF1^, elevated Bax/Bcl-2 ratio, and the activation of both intrinsic (mitochondrial-initiated) and extrinsic (Fas/FasL-initiated) apoptotic pathways. Furthermore, the p53 specific siRNA attenuated these effects. Conclusion: Our study suggested that ziyuglycoside I-triggered MDA-MB-231 cell cycle arrest and apoptosis were probably mediated by p53. This suggests that ziyuglycoside I might be a potential drug candidate for treating TNBC.

## 1. Introduction

Breast cancer is one of the most common malignant diseases. Among of all diagnosed cases, approximately 15%–20% are triple-negative breast cancer (TNBC) [1]. TNBC is a distinct pathological subgroup of breast cancer without estrogen receptor (ER), progesterone receptor (PR) and human epidermal growth factor receptor 2 (HER-2) [2]. Due to a lack of therapy targets, anthracycline- or platinum-based chemotherapy is the only systemic treatment option for TNBC patients at all stages [3,4]. However, resistance to chemotherapy is still the primary limiting factor against its effectiveness. It is particularly urgent to identify new therapeutic targets and develop novel regimens for TNBC with high efficiency and minimal side-effects.

The protein p53 has been recognized as an important tumor suppressor gene due to its functions in DNA repair, genomic stability, transcription, cell cycle control, and apoptosis [5,6]. During cell damage or cellular stress, p53 plays a key role in the quality control of both G1/S and G2/M cell cycle checkpoints to ensure the fidelity of DNA replication and repair [7]. Following DNA damage, p53 up-regulates cyclin-dependent kinase (CDK) inhibitor p21^WAF1^ level, which then triggers inhibition of CDK4,6/cyclin D, CDK2/cyclin E, and cyclin B1/Cdc2, respectively [8]. In addition, p53 also modulates both the intrinsic and the extrinsic apoptotic pathways. Activated p53 can trigger the intrinsic pathway by up-regulating the BH3-only family (such as Bax and NOXA) and down-regulating the Bcl-2 family members (such as Bcl-2 and Bcl-xL). Increased Bax/Bcl-2 ratio leads to the dysfunction of mitochondria by triggering mitochondrial outer membrane permeabilization and releasing cytochrome *c* into the plasma, which eventually causes caspase-mediated apoptosis [6]. In death receptor-initiated extrinsic apoptotic pathway such as Fas/FasL pathway, p53 can up-regulate the expression of both Fas and Fas ligands following DNA damage due to cytotoxic drug treatments [9]. Interestingly, a high occurrence of p53 mutations is observed in the vast majority of TNBCs [10,11]. Therefore, selecting drug candidates as to re-establish p53 pro-apoptotic function could be a novel approach in anti-TNBC therapy.

For decades, Chinese herbal medicine has been widely used in Asia as complementary or alternative medicines to anti-tumor agents. Over 80% of Chinese breast cancer patients used Chinese herbal medicines as adjuvant therapies [12]. The dried root of *Sanguisorba officinalis* L., *Radix Sanguisorbae*, is a widely used herbal product in China and Korea in treating inflammation, scalds, and internal hemorrhage [13]. Recent studies indicated that the extract of *Radix Sanguisorbae* also has anti-tumor effects on various cancers, including breast cancer [14,15,16]. However, the composition of *Sanguisorba officinalis* root extract is very complex; it is difficult to identify the particular ingredient(s) with anti-tumor effects. Previously, we have shown that ziyuglycoside II, one of the major components of *Radix Sanguisorbae*, has potent anti-tumor effects against breast cancer [17,18]. Little is known about the other components of *Radix Sanguisorbae* against cancers. Furthermore, understanding of the anti-tumor mechanisms of these components may provide novel insights into their potential applications in cancer therapy.

**Figure 1:**
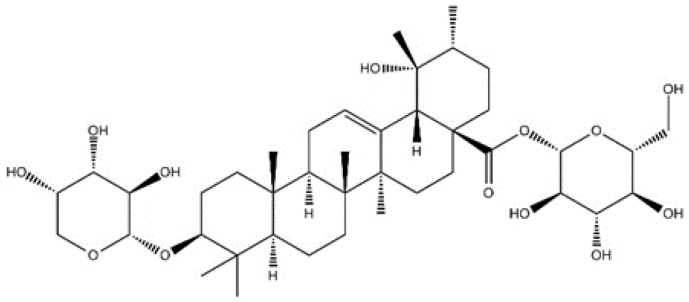
The chemical structure of ziyuglycoside I.

In the current study, we investigated the anti-tumor effect of ziyuglycoside I (another major component of *Radix Sanguisorbae*) (Figure 1) for the first time using triple-negative breast cancer MDA-MB-231 cells, and explored the molecular mechanisms underpinning this process.

## 2. Results

### 2.1. Ziyuglycoside I Suppressed the Proliferation of MDA-MB-231 Cells

**Figure 2:**
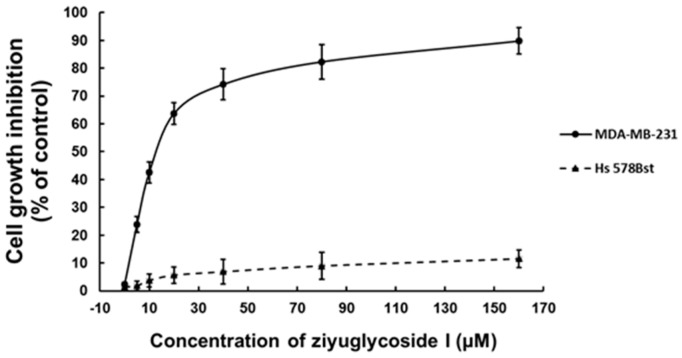
The inhibitory effect of ziyuglycoside I on MDA-MB-231 and Hs 578Bst cells proliferations. Both cell lines were treated with 5, 10, 20, 40, 80, and 160 µM of ziyuglycoside I for 24 h. Cell proliferation upon treatment was assessed by cell viability assay. All data were expressed as mean ± SE of three experiments and each experiment included triplicate repeats.

To investigate the growth inhibition effect of ziyuglycoside I, MDA-MB-231 and Hs 578Bst cells (normal control) were incubated with various concentrations of ziyuglycoside I for 24 h. Cell proliferation was subsequently measured by cell viability assay. Our results revealed that a marked anti-proliferation activity was observed in MDA-MB-231 cells after the treatment of ziyuglycoside I for 24 h with an IC_50_ value of 13.96 µM (Figure 2). Under the same experimental condition, ziyuglycoside I showed minimal cytotoxicity on the mammary epithelial cell line Hs 578Bst.

### 2.2. Ziyuglycoside I Induced G2/M Phase Arrest and Apoptosis on MDA-MB-231 Cells

**Figure 3:**
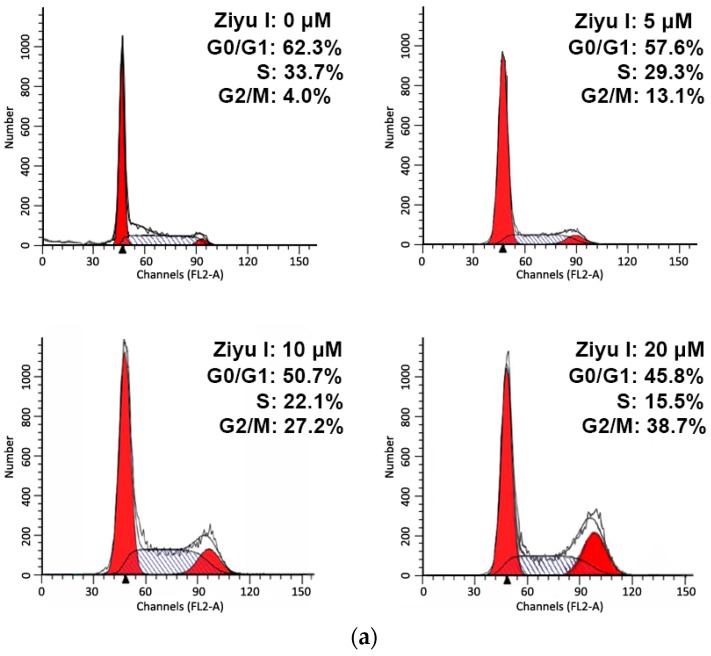

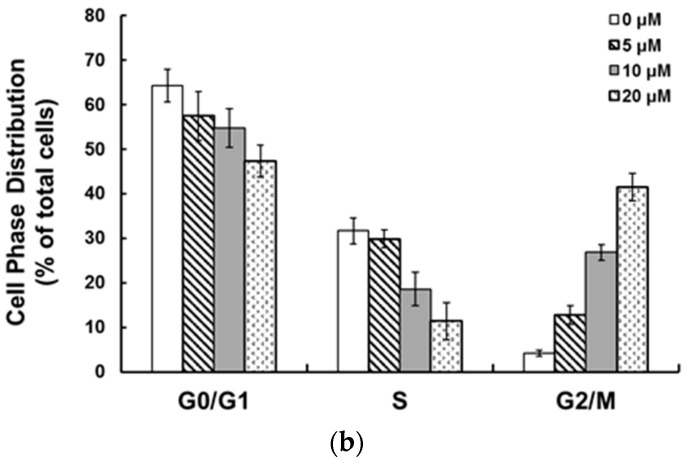
Cell cycle distribution of MDA-MB-231 cells with or without ziyuglycoside I treatment. The cells were treated with 5, 10, and 20 µM of ziyuglycoside I for 24 h. (**a**) Cell cycle distribution was analyzed by flow cytometry with PI-staining; (**b**) The data indicate the percentage of cells in different phases of the cell cycle with or without ziyuglycoside I treatment. All data were expressed as mean ± SE of three experiments and each experiment included triplicate repeats.

**Figure 4:**
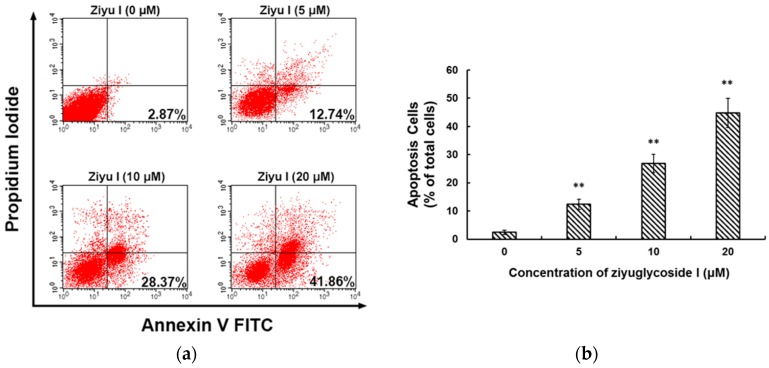
Apoptosis of MDA-MB-231 cells with or without ziyuglycoside I treatment. The cells were treated with 0, 5, 10, and 20 µM of ziyuglycoside I for 24 h. (**a**) Cell apoptosis was determined by flow cytometry with Annexin V/PI dual-staining; (**b**) The percentage of apoptotic cells with or without ziyuglycoside I treatment. All data were expressed as mean ± SE of three experiments and each experiment included triplicate repeats. ** *p* < 0.01 vs. control.

To determine whether the inhibition effect of ziyuglycoside I on MDA-MB-231 cell proliferations was due to cell cycle arrest or apoptosis, we performed flow cytometry analyses of PI- and annexin-V-stained cells. As shown in Figure 3, ziyuglycoside I induced a dose-dependent G2/M cell cycle arrest in MDA-MB-231 cells. Compared with the untreated cells, ziyuglycoside I treatment for 24 h caused a significant increase of the G2/M phase cells (4.17% ± 0.62%, 12.74% ± 2.08%, 26.77% ± 1.68%, and 41.38% ± 3.07% for 0, 5, 10, and 20 µM of ziyuglycoside I, respectively). The number of cells in the S phase also decreased with the treatment. We observed that ziyuglycoside I induced MDA-MB-231 cell apoptosis. After 24 h exposure to ziyuglycoside I (5, 10, and 20 µM), the percentage of apoptotic MDA-MB-231 cell was increased from 2.43% ± 0.79% to 12.37% ± 1.84%, 26.83% ± 3.21%, and 44.76% ± 5.17%, respectively (Figure 4).

### 2.3. Ziyuglycoside I Induced G2/M Phase Arrest in MDA-MB-231 Cells through Modulating Cell Cycle-Related Proteins

**Figure 5:**
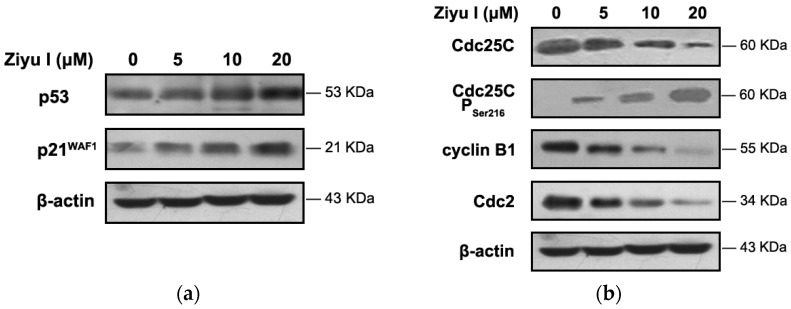
The effect of ziyuglycoside I on the expression of cell cycle-related proteins in MDA-MB-231 cells. Cells were treated with various concentrations of ziyuglycoside I (0, 5, 10, and 20 µM) for 24 h. Western blot analysis was adopted to assess the protein expression of. p53 and p21^WAF1^ (**a**) as well as that of several other cell cycle-related proteins (**b**). All data were representative of three independent experiments.

p53 protein, known as the “guardian of the genome”, mediates cell cycle arrest at major checkpoints [19]. Our results demonstrated that ziyuglycoside I treatment significantly increased the expression of p53. Activated p53 subsequently induced the expression p21^WAF1^, a potent cyclin-dependent kinase inhibitor (CKI), and led to G2/M phase arrest in MDA-MB-231 cells (Figure 5a). The cell cycle-related proteins in ziyuglycoside I-treated MDA-MB-231 cells were evaluated by Western blot. As shown in Figure 5b, following treatment, the level of phosphorylated Cdc25C at Ser216 was increased in a dose-dependent manner, while the expression of cyclin B1 and Cdc2 were significantly decreased.

### 2.4. Ziyuglycoside I Induced Apoptosis in MDA-MB-231 Cells through Intrinsic and Extrinsic Pathways

**Figure 6:**
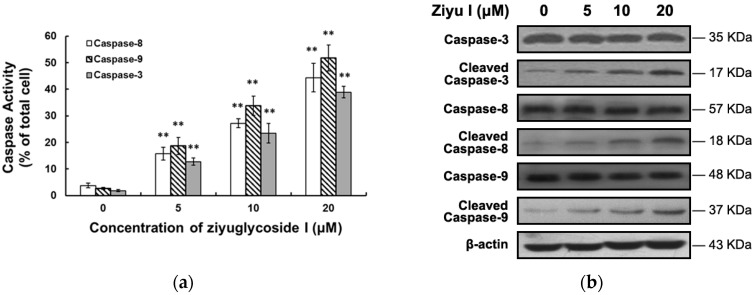
The effect of ziyuglycoside I on the activity and protein cleavage of caspases. Cells were exposed to various concentrations of ziyuglycoside I (0, 5, 10, and 20 µM) for 24 h. (**a**) The activity of caspase-8, caspase-9, and caspase-3 was determined as described in Materials and Methods. All data were expressed as mean ± SE of three experiments and each experiment included triplicate repeats. ** *p* < 0.01 vs. control; (**b**) The cleavage of caspase-8, caspase-9, and caspase-3 was assessed by Western blot.

Apoptosis is usually triggered by multi-signal pathways, in which caspase-mediated intrinsic and extrinsic pathways are most common [20]. The activities of two important initiators, caspase-8 and caspase-9, and their effector caspase-3, were investigated in our study. Ziyuglycoside I treatment pronouncedly increased the caspases activities in a dose-dependent manner (Figure 6a). As shown in Figure 6b, ziyuglycoside I could also induce the cleavage of caspas-8, caspase-9, and caspase-3. We then investigated whether the intrinsic and/or extrinsic pathways were involved in ziyuglycoside I-induced breast cancer cell apoptosis.

**Figure 7:**
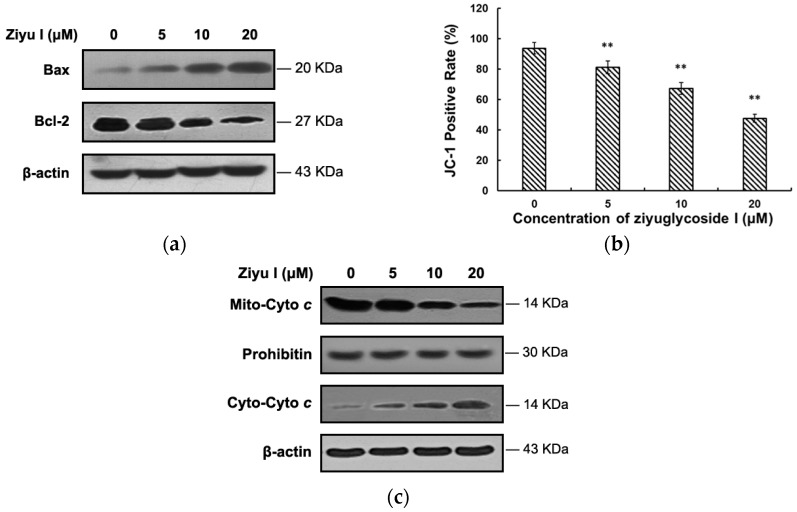
Ziyuglycoside I induced MDA-MB-231 apoptosis through the mitochondria-initiated intrinsic pathway. Cells were treated with various concentrations of ziyuglycoside I (0, 5, 10, and 20 µM) for indicated time. (**a**) The expression of Bax and Bcl-2; (**b**) Fluorescence ratio was used for MMP quantitative analysis; (**c**) The levels of mito and cyto cytochrome *c* were detected by Western blot analysis. All data were expressed as mean ± SE of three experiments and each experiment included triplicate repeats. ** *p* < 0.01 vs. control.

Ziyuglycoside I treatment up-regulated the pro-apoptotic proteins like Bax, and down-regulated anti-apoptotic proteins, such as Bal-2. Mitochondrial membrane potential was examined using fluorescent dye JC-1. Ziyuglycoside I treatment dose-dependently reduced the level of mitochondrial membrane potential (MMP) in MDA-MB-231 cells (Figure 7a), which led to an up-regulated release of cytochrome *c* from mitochondria to cytoplasm (Figure 7b). Results above demonstrated that the mitochondrial-initiated intrinsic pathway can be activated by ziyuglycoside I treatment in MDA-MB-231 cells.

**Figure 8:**
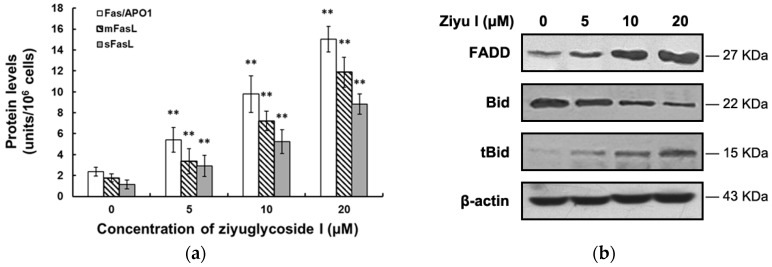
Ziyuglycoside I induced MDA-MB-231 apoptosis through the death receptor-initiated extrinsic pathway. Cells were treated with various concentrations of ziyuglycoside I (0, 5, 10, and 20 µM) for 24 h. (**a**) The expression levels of Aas/APO1 and FasL were determined by ELISA (Calbiochem, Danvers, MA, USA); (**b**) The expression of Bid and tBid were assessed by Western blot. All data were expressed as mean ± SE of three experiments and each experiment included triplicate repeats. ** *p* < 0.01 vs. control.

Caspase-8, a key protein in the extrinsic receptor-mediated pathway, was activated by ziyuglycoside I. Furthermore, we evaluated the expression of related proteins. As shown in Figure 8a, ziyuglycoside I treatment caused a dose-dependent up-regulation of both Fas/APO1 and FasL. Additionally, the expression of cell-membrane-bound FasL (mFasL) was higher than that of soluble FasL (sFasL). Activated Fas receptor in turn recruits Fas associated death domain (FADD), as well as promotes procaspase-8 and Bid self-cleavage. Ziyuglycoside I was shown to markedly induce the expression of FADD and the cleaved Bid (truncated Bid, tBid) in MDA-MB-231 cells (Figure 8b). These results identified that the Fas-initiated extrinsic pathway was also involved in ziyuglycoside I-induced MDA-MB-231 apoptosis.

### 2.5. Ziyuglycoside I-Induced Cell Cycle Arrest and Apoptosis in MDA-MB-231 Cells Were Partially Mediated by p53

**Figure 9:**
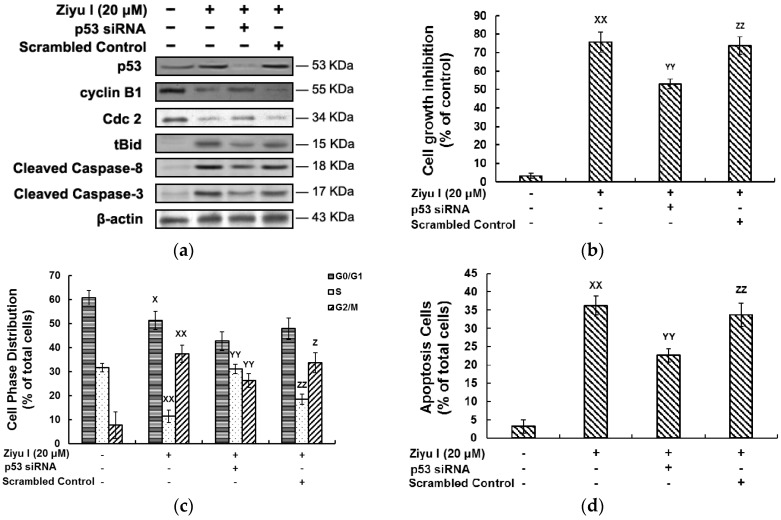
The involvement of p53 in ziyuglycoside I-induced cytotoxicity on MDA-MB-231 cells. Cells were transfected with p53 siRNA before exposure to ziyuglycoside I (20 µM) for 24 h. (**a**) The expression of cell cycle- and apoptosis-related proteins were assessed by Western blot; (**b**) The effect of p53 on ziyuglycoside I-induced inhibition of MDA-MB-231 cell proliferation; (**c**) The effect of p53 on ziyuglycoside I-induced MDA-MB-231 cell cycle arrest; (**d**) The effect of p53 on ziyuglycoside I-induced MDA-MB-231 cell apoptosis. All data were expressed as mean ± SE of three experiments and each experiment included triplicate repeats. “+”: with indicated treatment; “-“: without indicated treatment. ^X^ *p* < 0.05, ^XX^ *p* < 0.01 vs. control, ^YY^ *p* < 0.01 vs. Ziyu I (20 µM), ^Z^ *p* < 0.05, ^ZZ^ *p* < 0.01 vs. Ziyu I (20 µM) + p53 siRNA.

As described as the “guardian of the genome”, protein p53 plays a key role in the cell-cycle control and induction of apoptosis. To identify the role of p53 in ziyuglycoside I-induced G2/M phase arrest and apoptosis, MDA-MB-231 cells were transfected with p53-specific siRNA before exposure to ziyuglycoside I (20 µM). Western blot analysis showed a reduction of p53 protein level (Figure 9a). In p53 siRNA-transfected cells, ziyuglycoside I showed a lower cytotoxicity effect, compared with the control group (Figure 9b). Additionally, G2/M phase arrest and apoptosis induced by ziyuglycoside I treatment were significantly attenuated with the transfection of p53 siRNA (Figure 9c,d). The expressions of cell cycle and apoptosis related proteins were detected by Western blot. Ziyuglycoside I-induced reduction of cyclin B1 and Cdc2 were largely restored in p53 siRNA-transfected cells (Figure 9a). p53 silence also deactivated caspase, which mediates both intrinsic and extrinsic signaling pathways.

## 3. Discussion

Breast cancer, especially triple-negative breast cancer, is one of the most deadly malignant tumors in women in the world. Identifying novel therapeutic agents with low toxicity is highly demanding. In this study, we first reported the marked antiproliferative activity of ziyuglycoside I on triple-negative breast cancer MDA-MB-231 cells (IC_50_: 13.96 µM) with low cytotoxicity on normal mammary gland cells. Ziyuglycoside I treatment not only induced cell cycle arrest in G2/M phase, but also triggered apoptosis in MDA-MB-231 cells. Ziyuglycoside I was shown to activate both intrinsic and extrinsic pathways in these cells.

In cellular processes, checkpoint controls response to DNA repair and genome integrity protection [21]. p53 plays an important role in these processes by inducing cell cycle arrest and enhancing DNA repair. However, due to the mutations of p53 in breast cancer cells (including MDA-MB-231) [22,23], the G1 checkpoint is completely out of control and that of the G2/M checkpoint largely relies on checkpoint kinases, leading to infinite proliferation. Our study showed that ziyuglycoside I caused cell cycle arrest at the G2/M phase through up-regulating the expression of p53 and p21^WAF1^. Over-expression of p21^WAF1^ phosphorylates Cdc25C at Ser216, as well as inhibits the activity of cyclin B1/Cdc2. Phosphorylated Cdc25C provides a binding site for 14-3-3 proteins. The binding of 14-3-3 proteins to Cdc25C enables the nuclear export of this complex, deactivates Cdc2 [24].

Our results demonstrated that ziyuglycoside I treatment could trigger both mitochondria-initiated intrinsic pathway and Fas/FasL-initiated extrinsic pathway. These apoptotic signaling pathways have cross-talk among them. Stimulation of ziyuglycoside I in MDA-MB-231 cells activated the intrinsic pathway by up-regulating Bax/Bcl-2 ratio, releasing of cytochrome *c* from the mitochondrial intermembrane space, and activating caspase cascade. Meanwhile, in the extrinsic pathway, pro-caspase-8 activates the mitochondrial cytochrome *c* release by cleaving Bid [25], or triggers the downstream caspase effector directly via cleaved caspase-8. In both pathways, the activated initiator caspases mediate the activation of effector caspase-3, which is recognized as the key protease during apoptotic cell death [26]. Interestingly, caspase-3 not only acts as an effector but also amplifies the upstream death cascade. Conversely, over-expressed caspase-3 cleaves anti-apoptotic protein Bcl-2 converting to a Bax-like pro-apoptotic protein, and then triggers cell death by releasing cytochrome *c* from mitochondria [27,28].

p53 as an important tumor suppressor protein, is able to promote cell apoptosis via direct transcription-independent cellular signaling pathways [29]. As the most frequently mutated gene in breast cancer, identifying anti-mutant p53 agents are potentially useful in cancer therapy. Previous studies reported that a variety of compounds might restore wild-type p53 function by stabilizing the structure of mutant p53, or binding to mutant p53 proteins to promote proper folding of the mutant protein and, thus, restoring of p53 function [30]. We further investigated the role of p53 in ziyuglycoside I-induced MDA-MB-231 cytotoxicity. By blocking p53 with its specific siRNA, ziyuglycoside I-induced cell cycle arrest and apoptosis were partially attenuated. Moreover, the expression of crucial proteins in both intrinsic and extrinsic pathways were reversed. These results indicated that ziyuglycoside I-triggered MDA-MB-231 cell apoptosis was probably mediated by p53 protein. The mechanism underpinning the impact of ziyuglycoside I on mutant p53 is beyond the scope of this study, but will be further investigated in the future.

## 4. Materials and Methods

### 4.1. Materials

Ziyuglycoside I (≥98% purity, HPLC) was obtained from the National Institute for the Control of Pharmaceutical and Biological Products (Beijing, China). Dulbecco′s modified eagle′s medium (DMEM) and fetal bovine serum (FBS) were purchased from Gibco (Grand Island, NY, USA). 3-(4,5-dimethylthiazol-2-yl)-2,5-diphenyl tetrazolium bromide (MTT), propidium iodide (PI), 5,5′,6,6′-tetrachloro-1,1′,3,3′-tetraethylbenzimidazolylcarbocyanine (JC-1) were purchased from Sigma (St. Louis, MO, USA). FITC Annexin V Apoptosis Detection Kit I (BD556547) was obtained from BD Biosciences (San Jose, CA, USA). Caspase assay kits (ab39700 for caspase-8; ab65608 for caspase-9; ab39401 for caspase-3) and cytochrome *c* releasing apoptosis assay kit (ab65311) were obtained from Abcam (Cambridge, MA, USA). Dimethyl sulfoxide (DMSO), sodium bicarbonate, penicillin-streptomycin, trypsin, ribonuclease A (RNase A), polyvinylidenefluoride (PVDF) membrane and enhanced chemiluminescence (ECL) assay kit were purchased from Beyotime (Nantong, China). The information about the antibodies used in this study are shown as follows: p53 (1:2000, 10442-1-AP, Proteintech, Rosemont, IL, USA), p21 (1:1000, ab109520, Abcam), Cdc25C (1:1000, ab32444, Abcam), phospho-Cdc25C (Ser216) (1:1000, ab32051, Abcam), cyclin B1 (1:500, 647901, BioLegend, San Diego, CA, USA), Cdc2 (1:1000, #9112, CST, Danvers, MA, USA), Bax (1:2000, #2774, CST), Bcl-2 (1:1000, #15071, CST), caspase-8 (1:300, #9746, CST), caspase-9 (1:500, ab32539, Abcam), caspase-3 (1:500, #9662, CST), FADD (1:2000, sc-271520, Santa Cruz, Dallas, TX, USA), Bid (1:1000, #2002, CST), and the HRP conjugated goat anti mouse (1:5000, sc-2031)/rabbit (1:5000, sc-2030) secondary antibodies (Santa Cruz, Dallas, TX, USA).

### 4.2. Methods

#### 4.2.1. Cell Culture

Human breast carcinoma cell line MDA-MB-231 and human mammary gland cell line Hs 578Bst were obtained from the Shanghai Institute of Cell Biology, Chinese Academy of Sciences (Shanghai, China). MDA-MB-231 cells were cultured in DMEM medium supplemented with 10% FBS and 1% penicillin-streptomycin solution. Hs 578Bst cells were cultured in DMEM medium supplied with 30 ng/mL EGF, 10% FBS, and 1% penicillin-streptomycin solution. All of the cell lines were maintained at 37 °C in a humidified atmosphere containing 5% CO_2_.

#### 4.2.2. Cell Viability Assay

The effect of ziyuglycoside I on cell proliferation was examined by cell viability assay. Cells were seeded at density of 5 × 10^3^ cells/well in 96-well plates. After overnight cultured to confluence, cells were incubated with medium containing various concentrations of ziyuglycoside I (5, 10, 20, 40, 80, and 160 µM) for 24 h. Then, 20 µL of MTT (1 mg/mL) was added into each well and the plate was stayed at 37 °C for 4 h. The medium was then aspirated and DMSO was added for formazan solubilization. After gently shaking for 15 min, the absorbance of converted dye was measured at a wavelength of 562 nm using a fluorescence plate reader (SpectraMax M5, Molecular Devices, Sunnyvale, CA, USA). Three independent experiments were performed for each experimental condition.

#### 4.2.3. Cell Cycle Distribution

Cells (1 × 10^5^ cells/well) were seeded in six-well plates and serum starved for 24 h to synchronize into the G0 phase of cell cycle. Then cells were exposed to various concentrations of ziyuglycoside I (5, 10, and 20 µM) for 24 h, washed twice with ice-cold PBS, centrifuged and fixed in ice-cold 75% (*v/v*) ethanol for 1 h at 4 °C. After that, cells were suspended in propidium iodide (PI) solution (50 µg/mL) with ribonuclease A (RNase A) (0.1 mg/mL) for 30 min in the dark. Cell cycle distribution was assessed using flow cytometer (Becton-Dickinson, San Jose, CA, USA).

#### 4.2.4. Measurement of Cell Apoptosis

Apoptosis of cells was examined by double staining with Annexin V-FITC and PI according to the manufacturer′s protocols. Cells were seeded in six-well plates at a density of 2 × 10^6^ cells/well. After exposed to various concentrations of ziyuglycoside I (5, 10, and 20 µM) for 24 h, cells were collected and washed with PBS twice. Then the cells were resuspended into 500 µL binding buffer with 1.25 µL Annexin V-FITC and 10 µL PI, and incubated at room temperature for 15 min in the dark. After washing with binding buffer, the cells were analyzed with a flow cytometer. The Annexin V^+^/PI^−^ cells were considered as apoptotic cells and the percentage of which was calculated by CellQuest software (version 5.1, Becton-Dickinson).

#### 4.2.5. Caspase Activity Assay

Caspase activity was assessed according to the manufacturer′s instruction. Cells (1 × 10^6^) were resuspended in cell lysis buffer and incubated on ice for 30 min. Then the ice-cold cell mixtures were centrifuged at 10,000× *g* for 15 min and the suspensions were quantified. 50 µg protein of each sample was added into 96-well plate and incubated with different caspase specific substratesfor 1 h at 37 °C in the dark. Using a microtiter plate reader to detect the released pNA at OD405 for caspase activity.

#### 4.2.6. Measurement of Mitochondrial Membrane Potential

MDA-MB-231 cells were treated with various concentrations of ziyuglycoside I (5, 10, and 20 µM) for 6 h. Then cells were harvested and incubated in JC-1 (25 µM) for 30 min at 37 °C. Using flow cytometry, fluorescence changes in the ratio between the measurement at wavelengths of 590 nm (red) and 530 nm (green) fluorescence intensities indicated the alternation of Mitochondrial Membrane Potential (MMP) level.

#### 4.2.7. Measurement of Cytochrome *c* Release

Cells (5 × 10^5^ cells/well) were seeded in six-well plates and incubated with various concentrations of ziyuglycoside I (5, 10, and 20 µM) for 12 h. Cells were then collected by centrifugation at 600× *g* for 5 min at 4 °C, and washed with ice-cold PBS. Cells were resuspended with 1 mL Cytosol Extraction Buffer Mix containing dl-Dithiothreitol (DTT) (1 µM) and protease inhibitors, and incubated on ice for 10 min. Centrifuge at 10,000× *g* for 30 min at 4 °C, and the supernatant was collected as cytosolic fraction. 100 µL Mitochondrial Extraction Buffer Mix (containing 1 µM DTT and protease inhibitors) was added into the precipitate and vortex for 10 s. The product was saved as the mitochondrial fraction. The release of cytochrome *c* was evaluated by Western blot analysis as described below.

#### 4.2.8. Measurement of Fas/APO-1 and FasL Levels

The expressions of Fas/APO-1 and Fas ligand (FasL) including membrane Fas ligand (mFasL) of cell lysate and soluble Fas ligand (sFasL) of cell culture supernatant were assessed using ELISA kit (Calbiochem, Danvers, MA, USA). After incubation with ziyuglycoside I (5, 10, and 20 µM) for 24 h, the cells were incubated in 96-well plates with antibody cocktail at room temperature. Each well was then aspirated and washed to remove unbound materials and horseradish peroxidase conjugated streptavidin was added to each well. After catalyzing with chromogenic substrates, absorbance was measured at 450 nm with an ELISA reader. The expressions of Fas/APO-1 and FasL were determined by interpolating from standard curves.

#### 4.2.9. Transfection with Small Interfering RNA (siRNA)

MDA-MB-231 cells were plated in 100-mm dishes at a density of 2.5 × 10^6^ cells/dish. Cells were transfected with p53 siRNA (F: 5′-CACUACAACUACAUGUGUA-3′, R: 5′-UACACAUGUAGUUGUAGUG-3′) or scrambled control (F: 5′-CCUACGCCACCAAUUUCGU-3′, R: 5′-ACGAAAUUGGUGGCGUAGG-3′) siRNA using Lipofectamine 2000 (Invitrogen). After 48 h exposure to transfection mixture, cells were trypsinized and replated for the indicated experiments. Gene silencing efficiency was assessed by Western blot analysis after 48 h of transfection.

#### 4.2.10. Western Blot Analysis

After incubation with ziyuglycoside I (5, 10, and 20 µM) for the indicated time, cells were collected and lysed in ice-cold RIPA buffer for 30 min. Protein concentration was determined by the Bradford method [31]. 50 mg of protein from each sample was separated by 15% SDS-PAGE (sodium dodecyl sulfate-polyacrylamide gel electrophoresis). Gels were transferred electrophoretically on to PVDF membranes. PVDF membranes were then blocked with 5% non-fat dry milk in the mixture of TBST (Tris-Buffered Saline and Tween-20) for 1 h, and incubated with primary antibodies at 4 °C for 12 h. Membranes were washed with TBST and incubated with corresponding secondary antibody at room temperature for 1 or 2 h. The bands on the membranes were visualized using the ECL assay kit. The density of each band was normalized to the expression of β-actin.

#### 4.2.11. Statistical Analysis

Biostatistical analyses were conducted using the SPSS 19.0 software package (IBM Analytics, Armonk, NY, USA). Quantitative data were expressed as mean ± SE. Statistical comparisons of multiple groups were performed with a one-way ANOVA followed by Tukey′s *post hoc* test and *p* < 0.05 was considered as significant.

## 5. Conclusions

**Figure 10:**
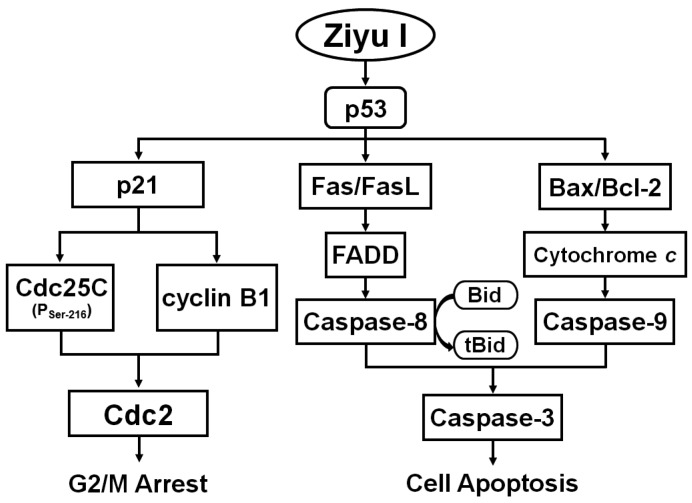
The proposed molecular mechanisms of ziyuglycoside I-mediated cell cycle arrest and apoptosis in MDA-MB-231 cells.

In conclusion, our study indicated that ziyuglycoside I inhibited the proliferation of triple-negative breast cancer MDA-MB-231 cells by inducing p53-mediated G2/M arrest and intrinsic/extrinsic apoptotic pathways (Figure 10). The cytotoxic effects of ziyuglycoside I against breast cancer cells contributes to understanding the molecular mechanism of the Chinese herbal medicine, *Radix Sanguisorbae*, as an adjuvant anti-cancer agent. Our study provided theoretical and experimental evidence to the development based on Chinese herbal medicine in breast cancer treatment.
